# optiGAN: a deep learning-based alternative to optical photon tracking in Python-based GATE (10+)

**DOI:** 10.1088/1361-6560/ade2b5

**Published:** 2025-07-02

**Authors:** Guneet Mummaneni, Carlotta Trigila, Nils Krah, David Sarrut, Emilie Roncali

**Affiliations:** 1 Department of Computer Science, University of California, Davis, Davis, CA, United States of America; 2 Department of Biomedical Engineering, University of California, Davis, Davis, CA, United States of America; 3 Université de Lyon; CREATIS; CNRS UMR5220; Inserm U1294; INSA-Lyon, Université Lyon 1, Lyon, France; 4 Department of Research and Development, Holland Proton Therapy Centre Delft, Delft, The Netherlands

**Keywords:** optical photon transport, generative adversarial network, Monte Carlo simulation, GATE 10, deep learning acceleration, multidimensional distributions

## Abstract

*Objective.* To accelerate optical photon transport simulations in the GATE medical physics framework using a generative adversarial network (GAN), while ensuring high modeling accuracy. Traditionally, detailed optical Monte Carlo methods have been the gold standard for modeling photon interactions in detectors, but their high computational cost remains a challenge. This study explores the integration of optiGAN, a GAN model into GATE 10, the new Python-based version of the GATE medical physics simulation framework released in November 2024. *Approach.* The goal of optiGAN is to accelerate optical photon transport simulations while maintaining modeling accuracy. The optiGAN model, based on a GAN architecture, was integrated into GATE 10 as a computationally efficient alternative to traditional optical Monte Carlo simulations. To ensure consistency, optical photon transport modules were implemented in GATE 10 and validated against GATE v9.3 under identical simulation conditions. Subsequently, simulations using full Monte Carlo tracking in GATE 10 were compared to those using GATE 10–optiGAN. *Main results.* Validation studies confirmed that GATE 10 produces results consistent with GATE v9.3. Simulations using GATE 10–optiGAN showed over 92% similarity to Monte Carlo-based GATE 10 results, based on the Jensen–Shannon distance across multiple photon transport parameters. optiGAN successfully captured multimodal distributions of photon position, direction, and energy at the photodetector face. Simulation time analysis revealed a reduction of approximately 50% in execution time with GATE 10–optiGAN compared to full Monte Carlo simulations. *Significance.* The study confirms both the fidelity of optical photon transport modeling in GATE 10 and the effective integration of deep learning–based acceleration through optiGAN. This advancement enables large-scale, high-fidelity optical simulations with significantly reduced computational cost, supporting broader applications in medical imaging and detector design.

## 1. Introduction

Optical photon transport simulations play a crucial role in various scientific and engineering domains, particularly in medical imaging, high-energy physics, and radiation detection. In nuclear imaging including positron emission tomography (PET) scanner simulations, accurate modeling of optical photon transport is essential for scintillation detector response characterization, light collection efficiency estimation, and energy and time resolution analysis (Roncali and Cherry 2013, Berg *et al* 2015).

Optical photon transport simulations also contribute significantly to clinical advances in imaging technology. For example, Berg *et al* (2015) used these simulations to optimize light transport in scintillation crystals for time-of-flight PET, contributing to improvements in timing resolution, a key performance factor in clinical PET systems. In another study, Berg *et al* (2016) demonstrated the design of a combined time-of-flight and depth-of-interaction PET detector for total-body imaging, supported by these simulations to evaluate detector performance. Together, these studies underscore the role of optical photon transport simulations as a foundational tool for designing detectors that ultimately impact clinical imaging quality.

Traditionally, Monte Carlo methods have been the gold standard for simulating optical photon transport, as they accurately model stochastic photon interactions, including scattering, absorption, and reflections at material boundaries. These boundaries include scintillator surfaces in contact with a reflector, or interfaces with a photodetector such as a silicon photomultiplier (SiPM).

Even though significant efforts have been made in open-source Monte Carlo simulation toolkits to support multi-threading on the CPU or GPU for improved simulation speed (Bert *et al* 2012, Bert *et al* 2013), Monte Carlo simulations remain computationally expensive, often requiring hours to days to generate sufficient photon statistics for high resolution imaging and detector optimization. This computational cost underscores the interest in deep learning-based approaches to accelerate simulations.

Recent efforts to accelerate Monte Carlo simulations (de Oliveira *et al* 2017, Paganini *et al* 2018, Erdmann *et al* 2019, Sarrut *et al* 2019, Sarrut *et al* 2021b, Fanelli and Pomponi 2020, Hashemi *et al* 2023) have adopted generative adversarial networks (GANs, Goodfellow *et al* 2014) thanks to their ability to learn complex data distributions and generate realistic synthetic data. A GAN consists of two neural networks, a generator that produces synthetic samples and a discriminator that evaluates whether the samples are real or generated. Through an adversarial training process, both networks improve iteratively, enabling the generator to create increasingly realistic data. Unlike traditional neural networks, which rely on direct supervised learning, GANs excel at capturing complex, high-dimensional distributions, making them well-suited for problems where traditional interpolation methods fall short. In the context of optical photon transport, GANs can efficiently model the intricate probabilistic nature of photon interactions, offering a computationally efficient alternative to Monte Carlo methods.

This approach was successfully applied to optical photon transport by (Trigila *et al* 2023), where a GAN-based model was trained on a multidimensional dataset comprising optical photon transport parameters at the photodetector face (e.g. position, direction, energy), generated using the GATE (Geant4 Application for Tomographic Emission) toolkit (Sarrut *et al* 2021a). The model utilized emission spatial coordinates as conditional labels, allowing it to generate physically realistic photon distributions. The results demonstrated that the GAN model accurately learned the underlying data distributions, validating its potential to accelerate optical photon transport simulations while maintaining accuracy.

To enable real-time detector simulation using optiGAN, direct integration into GATE is necessary.

GATE is one of the most widely used toolkits for numerical simulations in medical imaging, making it an essential platform for integrating optiGAN to ensure accessibility for the broader research community.

The openGATE collaboration has been actively developing GATE for 20 years, initially focusing on nuclear imaging (Santin *et al* 2003, Strulab *et al* 2003, Jan *et al* 2004, Buvat and Lazaro 2006) and later expanding to applications such as external and internal radiotherapy (Jan *et al* 2011), dosimetry (Sarrut *et al* 2014), and hadron therapy (Grevillot *et al* 2020). Earlier versions of GATE, prior to GATE 10, were developed in C++ and introduced a macro-scripting interface, enabling users to efficiently define and run simulations. However, these versions were not well suited for machine learning integration, limiting their ability to utilize modern data-driven simulation techniques.

Recently, significant efforts have been made to develop GATE 10, a new version of GATE entirely based on Python (Krah *et al* 2024). In this latest release (openGATE 2024), the entire code, except for certain core classes, was rewritten in Python. This refactoring introduced a major shift from previous versions (e.g. v.9.3), enabling users to write simulations directly in Python. This transition enhances integration with data analysis and machine learning frameworks, as most modern computational tools are Python-compatible, making GATE 10 more agile and suitable for advanced simulation workflows.

In this work, the major code revision in the newly released GATE 10 necessitates, as an initial step, validating that it produces the same results as GATE 9 before integrating optiGAN into GATE 10. While the generation and tracking of optical photons are entirely handled by the Geant4 C++ backend, several front-end components required to configure and enable optical photon transport components, which were previously not implemented in GATE 10, were first integrated to enable optical simulations. With these components in place, the complete pipeline for generating output using optiGAN was then incorporated into GATE 10. In the rest of the paper, GATE 10 with optiGAN is called GATE 10–optiGAN and GATE 10 without optiGAN is simply called GATE 10.

**Figure 1.**
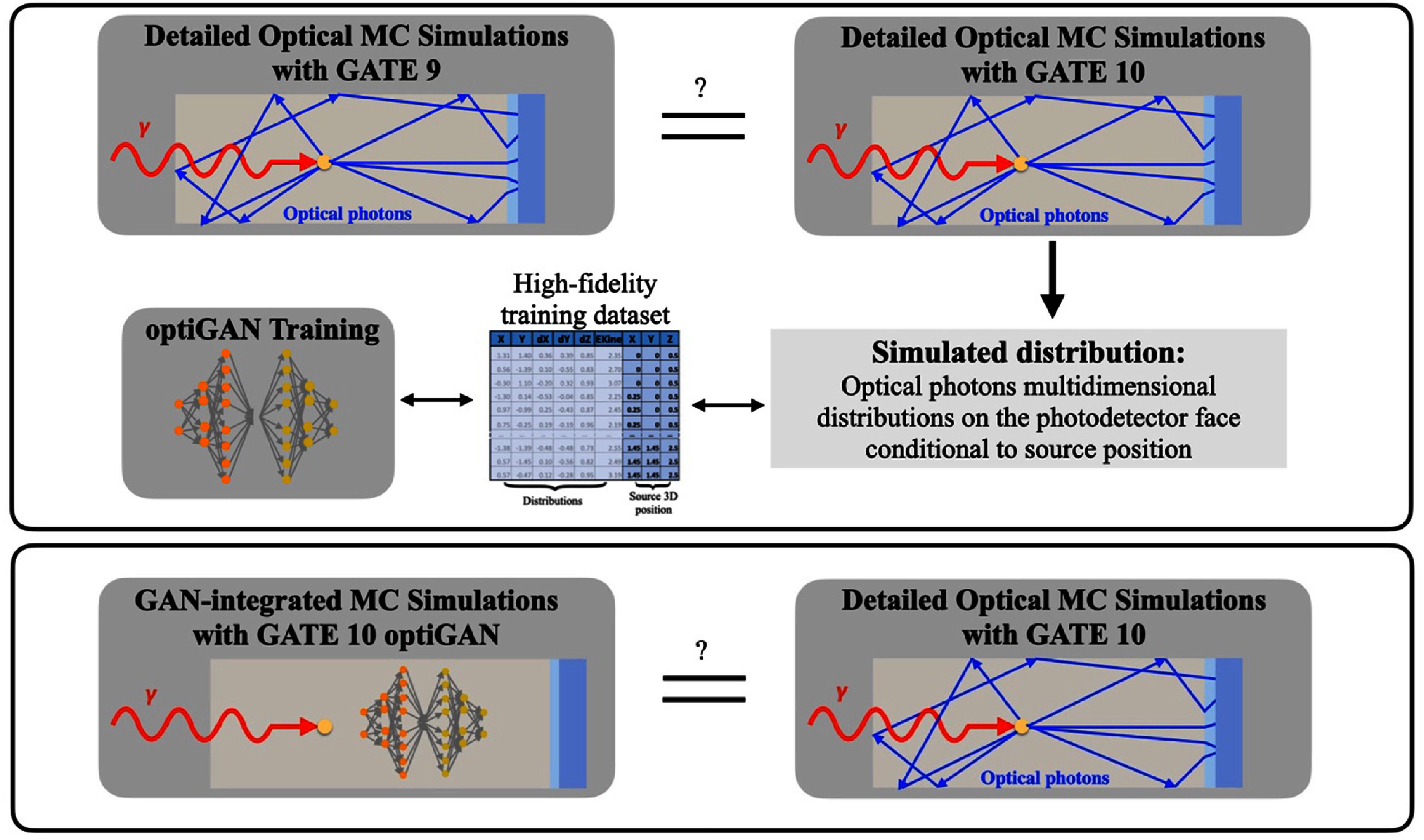
Schematic of this work pipeline. First, GATE 9 and GATE 10 full optical Monte Carlo simulations were performed and compared in terms of similarity. GATE 10 high-fidelity simulations were used to create the training dataset for the optiGAN model. The best-performing model checkpoint was integrated directly into GATE 10 pipeline. GATE 10 and GATE 10–optiGAN simulations were then compared in terms of similarity.

To evaluate the accuracy of the implementation, a set of multidimensional distributions of optical photons at the photodetector face was generated using GATE 9 and GATE 10 (figure 1, top row). Identical simulation setups were used to ensure a valid comparison with a bismuth germanate (BGO) crystal as example.

The data from GATE 10 simulations were used to build a training dataset for optiGAN (figure 1, top row), ensuring that the model learned the statistical distributions of optical photons at the photodetector face. Once the training was completed, the best-performing checkpoint was used in GATE 10–optiGAN, enabling the generation of synthetic optical photon data directly within the simulation framework (figure 1, bottom row).

To assess the accuracy and reliability of the GATE 10–optiGAN, the same set of emission points used in the training dataset was simulated using this pipeline and results were compared with those from GATE 10 simulations.

Since GATE 10–optiGAN simulations are expected to be computationally more efficient than the Monte Carlo method, a detailed computational time analysis was conducted.

## 2. Materials and methods

### 2.1. Monte Carlo GATE 9 simulation setup

**Figure 2.**
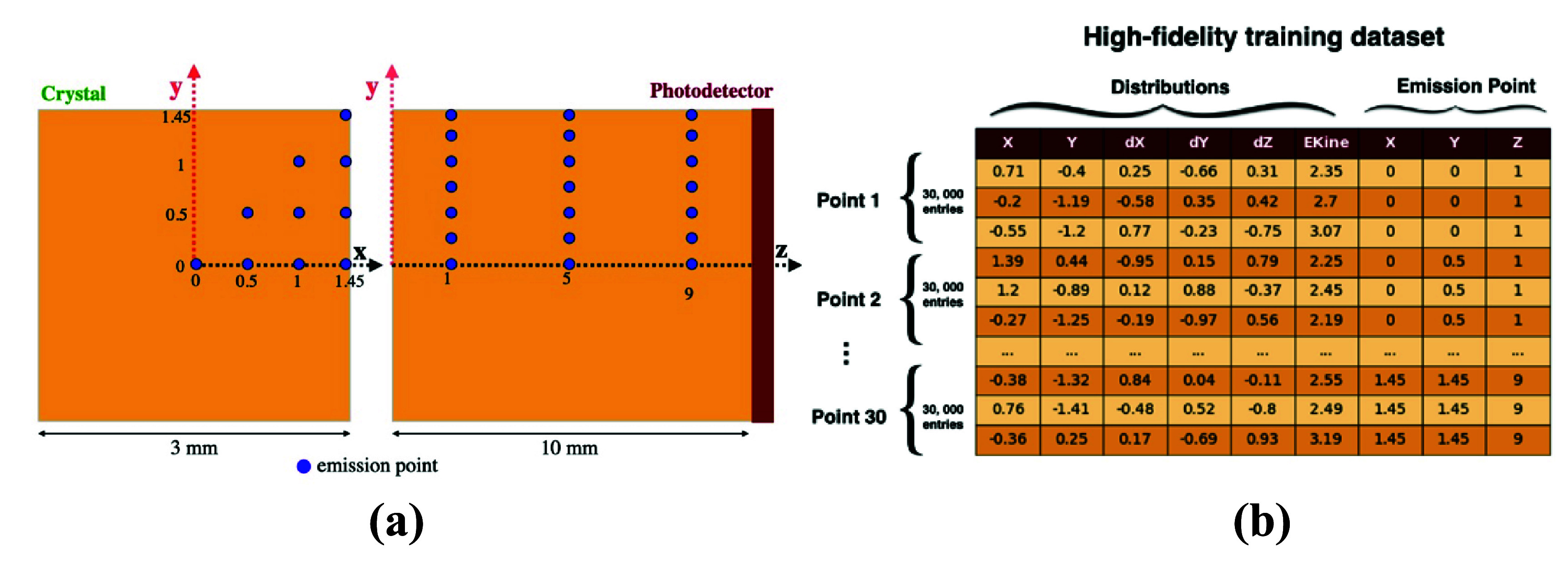
(a) Overview of the training dataset used for high-fidelity optical photon simulation. (b) Structure of the training dataset associated with each emission point. Each row represents a photon emission event, characterized by spatial coordinates (*X, Y*), directional components (*dX, dY, dZ*), kinetic energy (EKine), and emission coordinates (*X, Y, Z*).

The simulation in GATE v9.3 (Sarrut *et al* 2021a) was performed using a 3 × 3 × 10 mm^3^ BGO crystal as the scintillator. A 420 keV electron source was used to initiate the simulation. The energy of 420 keV corresponds to the energy of photoelectrons created by 511 keV gamma photon interactions in BGO. Interactions of electrons and subsequent generation and transport of optical photons were modeled using the electromagnetic physics model from Geant4 (Geant4 Collaboration 2021). Specifically, we used the emstandard_opt4 physics list provided by Geant4, which offers optimized accuracy for low-energy electromagnetic processes. A custom energy range cut of 10 *µ*m was applied to ensure accurate modeling of electron and photon interaction within the BGO crystal. This model, based on detailed electromagnetic interactions, accurately describes low-energy electron interactions, including ionization, and multiple scattering effects, ensuring realistic optical photon generation within the scintillator. Using an electron source instead of randomly distributed gamma interactions allowed us to control precisely the emission position of optical photons. A total of 30 different points within the crystal volume were simulated (figure 2(a)). These positions covered 1/8 of the crystal cross-section (ten points per cross-section) and spanned three depths of interaction, following the approach of (Trigila *et al* 2023).

To simulate the boundary conditions of the crystal, a customized lookup table model (LUT Davis model) was used (Roncali and Cherry 2013, Roncali *et al* 2017b). The LUT accurately accounts for the optical transport at the material interfaces, including internal reflections and refraction effects, providing precise modeling of photon transport at the crystal boundaries and was validated against experiments (Roncali *et al* 2017b, Trigila *et al* 2024).

Five faces of the BGO crystal were surrounded by Teflon to provide high reflectivity, enhancing light collection efficiency, while the photodetector face was optically coupled using Epoxy with an index of refraction of 1.56. This optical interface facilitates efficient light transmission by reducing the refractive index mismatch between the BGO crystal and the silicon dioxide (SiO2) photodetector, thereby minimizing internal reflection losses. All crystal surfaces were modeled using customized LUTs (e.g. crystal-reflector and crystal-epoxy) (Trigila *et al* 2021).

To focus on the optical transport within the scintillator, the photodetector was considered ideal with a photodetection efficiency of 1, ensuring that every optical photon reaching the detector was detected. This assumption eliminated any variations due to detector efficiency, allowing for a direct comparison of optical photon transport between GATE 9, GATE 10, and GATE 10–optiGAN.

The output of GATE 9 simulations was recorded using a Phase Space Actor, which stored detailed information about each particle that entered a specific volume, including its position, direction, energy, and timing throughout the simulation in a ROOT file. While the actor provided a wide range of attributes describing the full phase space of optical photons, the analysis for this study focused specifically on the spatial coordinates (*X, Y*), momentum components (*dX, dY, dZ*), and kinetic energy (EKine) of the optical photons arriving at the photodetector face (figure 2(b)). These parameters are crucial for understanding optical photon transport, as they define the particle’s trajectory and energy evolution within the system.

### 2.2. Monte Carlo GATE 10 simulation setup

During its initial development, GATE 10 lacked several key components of optical transport, including optical photon generation, optical surface boundary creation, and the integration of material and surface properties. These missing components were integrated into GATE 10 to ensure full functionality. A comparison with GATE 9 was conducted as a validation step to confirm that all aspects of optical photon transport were functioning as intended (figure 1, top row). Additionally, the validated optical photon transport data from GATE 10 was used as the training dataset for optiGAN.

To enable a direct comparison of optical photon transport, the GATE 10 simulation setup was identical to that of GATE 9.

The GATE 10 output was saved using a Phase Space Actor, which stored optical photon parameters (*X, Y, dX, dY, dZ*, and EKine) at the photodetector face. These distributions were generated for all 30 emission coordinates and combined into a multidimensional matrix, which was then used to train optiGAN.

### 2.3. Integration of optiGAN in GATE 10

The next step involved training the optiGAN model and then integrating it into GATE 10.

#### 2.3.1. optiGAN model

In optiGAN, a Wasserstein GAN (Arjovsky *et al* 2017) with gradient penalty (GP) (Gulrajani *et al* 2017) combined with a Conditional GAN (Mirza 2014) was implemented. The WGAN framework optimizes the Wasserstein distance, improving gradient propagation and reducing mode collapse, while the GP enforces the 1-Lipschitz constraint without the need for weight clipping. The Conditional GAN component ensures that the generated optical photon transport data adheres to physical constraints by conditioning the generation process on specific input parameters. Following the WGAN formulation, we refer to the discriminator as critic from this point forward.

The model was trained on all optical photon transport parameters conditioned on their emission coordinates, resulting in a dataset of 900k rows. Each of the 30 emission coordinates within the 3 × 3 × 10 mm^3^ crystal produced 30 000 entries, leading to the total dataset size (generated by the simulation described in 2.2). During training, the dataset was normalized, and the mean and standard deviation of each feature were stored for denormalization of the output data.

The generator was conditioned on the emission point coordinates (class*X*, class*Y*, class*Z*), allowing it to generate photon trajectories that align with real simulation distributions. This conditional approach ensured that optiGAN effectively learned the complex dependencies between emission points and photon transport dynamics, improving the accuracy of the generated data.

The model architecture remained largely similar to that proposed by Trigila *et al* (2023), Srikanth *et al* (2024), with the addition of an extra layer in both the generator and critic, which experiments demonstrated improved training accuracy (not shown here). The generator G followed a layer configuration of H, 2 H, 2 H, 4 H while the critic followed a 4 H, 2 H, 2 H, H structure. The value of H was empirically set to 128. Following the recommendations of Arjovsky *et al* (2017), ReLU activation was applied in all hidden layers, except in the final layer of the generator, where a linear activation function was used.

The model was implemented and trained using PyTorch, following an update ratio of 3 and 1 for the critic and generator respectively. Both the generator and the critic were optimized using the Adam optimizer, with its hyperparameter ${\beta _1}$ and ${\beta _2}$ set to 0.5 and 0.9, and a learning rate of $5 \times {10^{ - 5}}$. The training was conducted for 116k epochs. Model checkpoints were saved after every epoch, and the best checkpoint, indicating the epoch where the generated and real distribution were the most similar, was selected for integration into GATE 10.

#### 2.3.2. GATE 10–optiGAN simulation setup

The integrated optiGAN model requires the number of optical photons (${n_{{\text{op}}}}$), which determines the number of outputs, along with the emission coordinates (due to conditional training approach) as inputs to generate synthetic optical photon transport data. Usually, in GATE 10 simulations, this information can be found in the ROOT file generated by the Phase Space Actor. Since the objective was to replace the computationally intensive Monte Carlo-based optical photon transport with optiGAN, an alternative method was required to determine the number of optical photons without explicitly simulating their transport.

One possible approach involved modifying Geant4 classes to extract this information; however, this quickly became impractical due to the intricate class hierarchy. Furthermore, this approach would have required GATE 10 users to wait for the next Geant4 release to incorporate these modifications, potentially introducing significant delays. Modifications were thus applied only within GATE 10, enabling the direct extraction of the necessary information.

A GATE 10 feature called *KillActor* was added to the setup to selectively terminate specified particles. In this implementation, the *KillActor* was configured to eliminate optical photons after their first step, effectively terminating them upon reaching the boundary surface of the crystal. This is particularly important because each optical photon typically undergoes hundreds to thousands of interactions within the scintillator due to scattering, absorption, and reflections before reaching a photodetector, making full photon tracking computationally expensive.

To retain essential particle data, the *KillActor* was coupled with the PhaseSpace Actor, allowing relevant information to be stored until the particles were terminated. With this approach optical photons were created within the simulation, providing the required ${n_{{\text{op}}}}$, but were promptly discarded before undergoing further transport.

The second input required by optiGAN, the emission coordinates, was recorded by the PhaseSpace Actor, which was attached to the photodetector volume and stored in a ROOT file during the simulation. Since the PhaseSpace Actor logs data for all particles in the simulation, extracting the emission coordinates (corresponding to the electron location), was necessary. To achieve this, the first recorded electron position for each event was selected as the emission coordinates.

Both the number of optical photons ${n_{{\text{op}}}}$ and the electron emission position were then used as input for the trained generator model to produce the feature outputs. The generated data was denormalized using the statistical parameters saved during training to restore it to its original scale. Finally, the processed output was saved in a ROOT file, allowing for easy import and analysis.

### 2.4. Comparison methodology

The objective was to compare GATE 9, GATE 10, and GATE 10–optiGAN approaches. A fixed number of 30 000 photon entries was used per emission point in GATE 9 and 10. However, in GATE 10–optiGAN, the number of optical photons generated varied depending on the electron activity and the number of optical photons emitted per electron.

For example, a simulation conducted with an electron activity level of 1000 Bq, resulted in approximately 2.5 million optical photons for all events. To ensure a fair comparison between methods, a subset of 30 000 photon outputs obtained from the GATE 10–optiGAN simulation was randomly sampled to match the dataset size used in GATE 9 and GATE 10, allowing for a direct assessment of performance and accuracy.

To evaluate the similarity between the GATE 10—optiGAN simulation distributions and GATE 10 distributions, Jensen–Shannon (JS) distance (JSD) was selected as the primary metric due to its symmetric nature, and its ability to provide a reliable measure of divergence between probability distributions. It is also bounded between [0,1], making it practical to extract percentages values. The similarity was finally estimated as 1-JSD.

### 2.5. Computation time

The computational time difference between GATE 10 and the GATE 10–optiGAN simulations was evaluated. To achieve this, *py-spy*, an open-source Python program profiler, was used to generate a detailed flame chart, providing a visualization of computation speed and execution time.

*Py-spy* enabled real-time performance monitoring without requiring modifications to the code or restarting the program. With minimal overhead, it ensured that performance profiling did not interfere with execution. These features made *py-spy* particularly well suited for this study, as profiling GATE 10 simulations, which involved complex physics calculations and long execution times, required a nonintrusive, high-performance profiling tool that did not affect the simulation’s behavior or accuracy.

To assess performance across different simulation conditions, both GATE 10 and GATE 10–optiGAN simulations were conducted over activity levels ranging from 10 to 3000 Bq, with emission coordinates set to [0.5, 0.5, 1.0] mm, which was selected from the training dataset.

All simulations, model training, inference, and computation time estimations were conducted on an Apple M4 Pro, featuring a 12-core CPU, 16-core Neural Engine, and 48GB Unified Memory. The training process was performed on a GPU to optimize computational efficiency.

## 3. Results

### 3.1. GATE 9 and GATE 10 similarity

**Figure 3.**
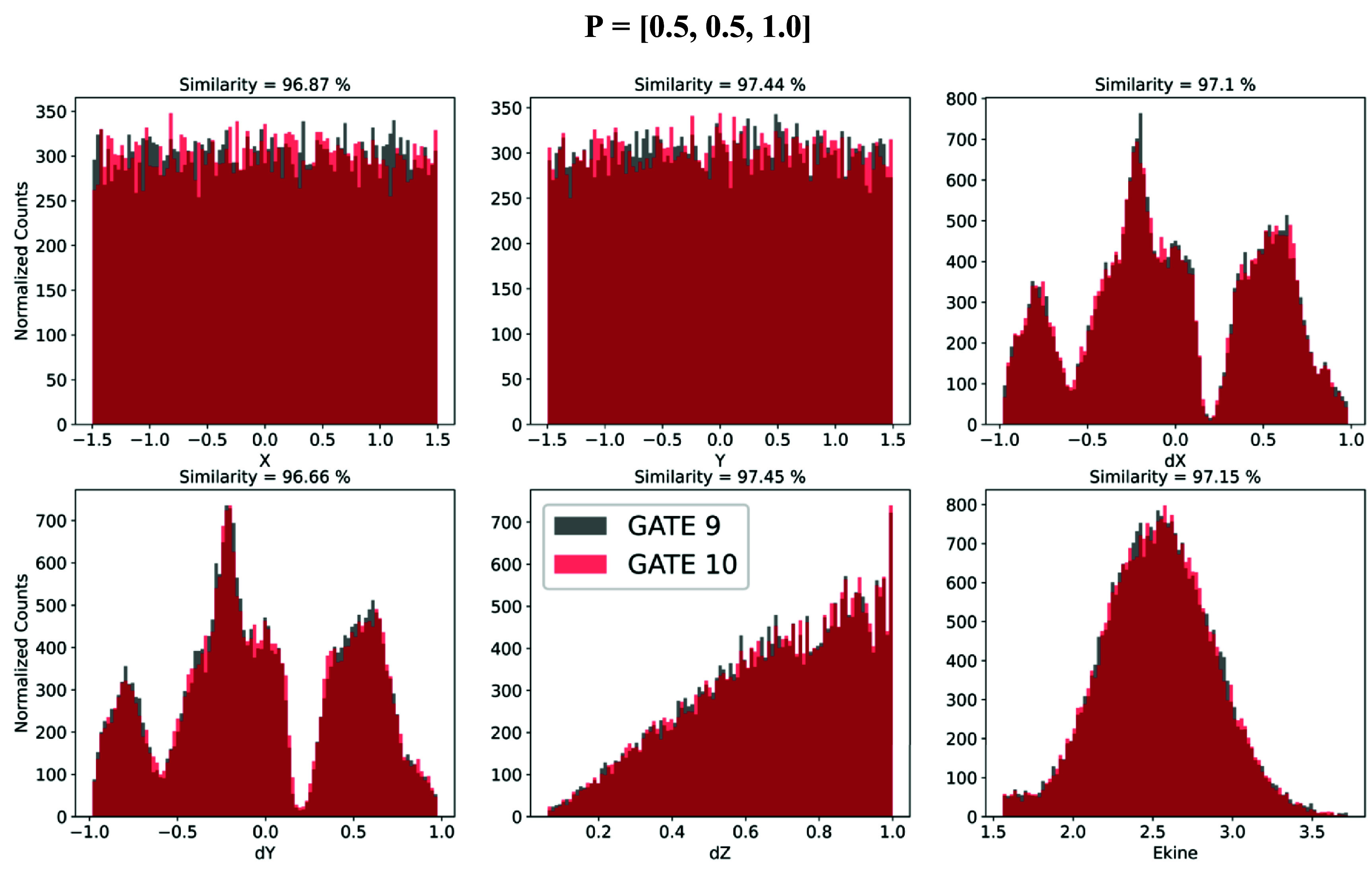
Comparison of GATE 9 and GATE 10 simulations for optical photon transport. The plots show the normalized distributions of position (*X, Y*), direction (*dX, dY, dZ*) and kinetic energy for optical photons at emission position *P* = [0.5, 0.5, 1.0]. GATE 9 results are shown in black and GATE 10 in red.

The illustrative point shown in figure 3 indicates that GATE 9 and GATE 10 simulations are in excellent agreement with similarity values greater than 96.7% for all features. The close agreement between the two simulations indicates that all previously missing modules were successfully incorporated, preserving the accuracy of optical photon transport within the updated framework of GATE 10.

**Figure 4.**
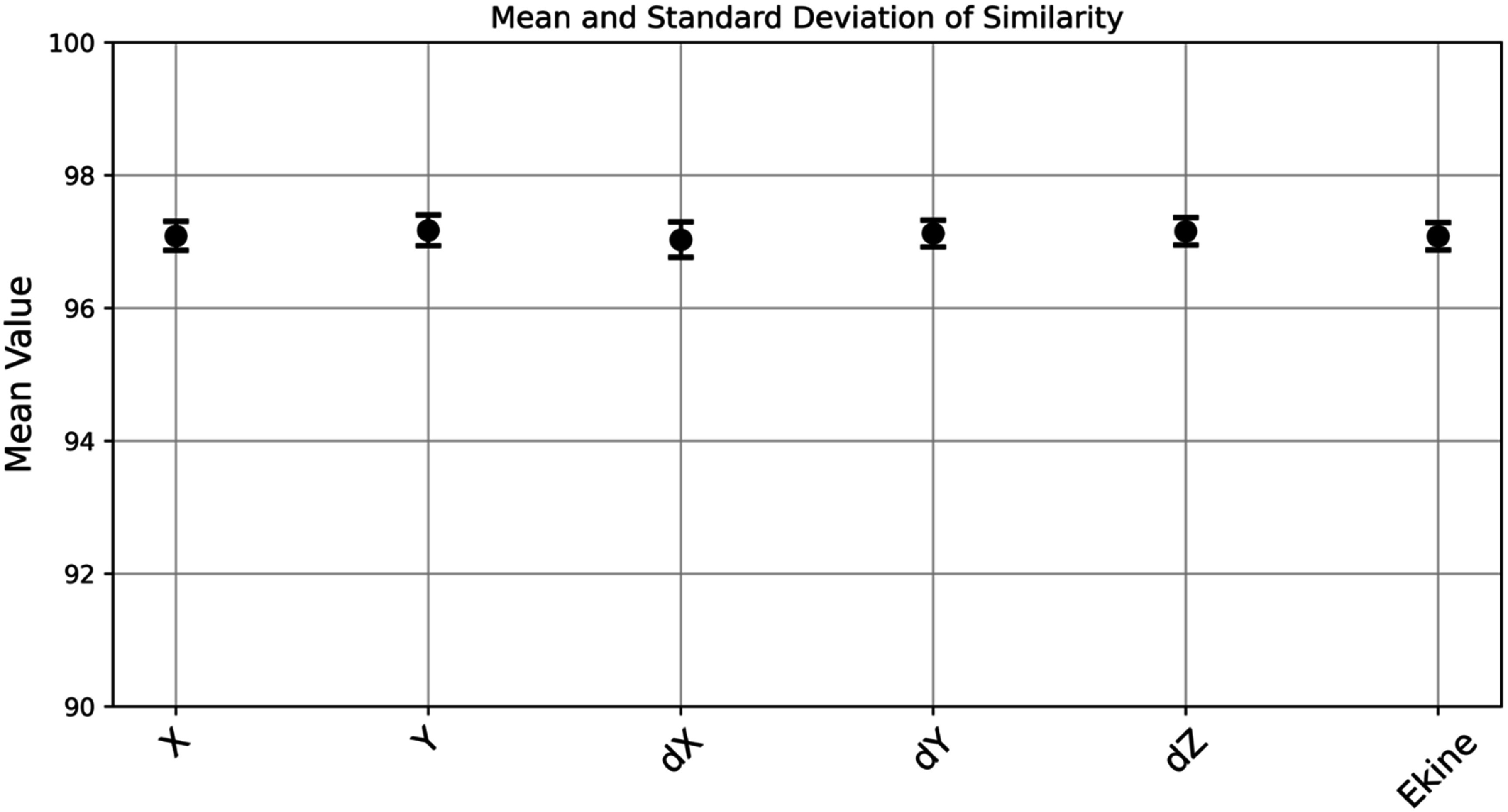
Mean and standard deviation of similarity between GATE 9 and GATE 10 simulations for all emission coordinates within a 3 × 3 × 10 mm^3^ scintillation crystal.

Further validation is presented in figure 4, which shows the mean similarity scores (∼97%) along with their standard deviations for all 30 emission coordinates chosen for the dataset across different photon transport parameters.

Next, standard GATE 10 simulations and the GATE 10–optiGAN simulations were compared to evaluate optiGAN.

### 3.2. GATE 10 and GATE 10–optiGAN similarity

**Figure 5.**
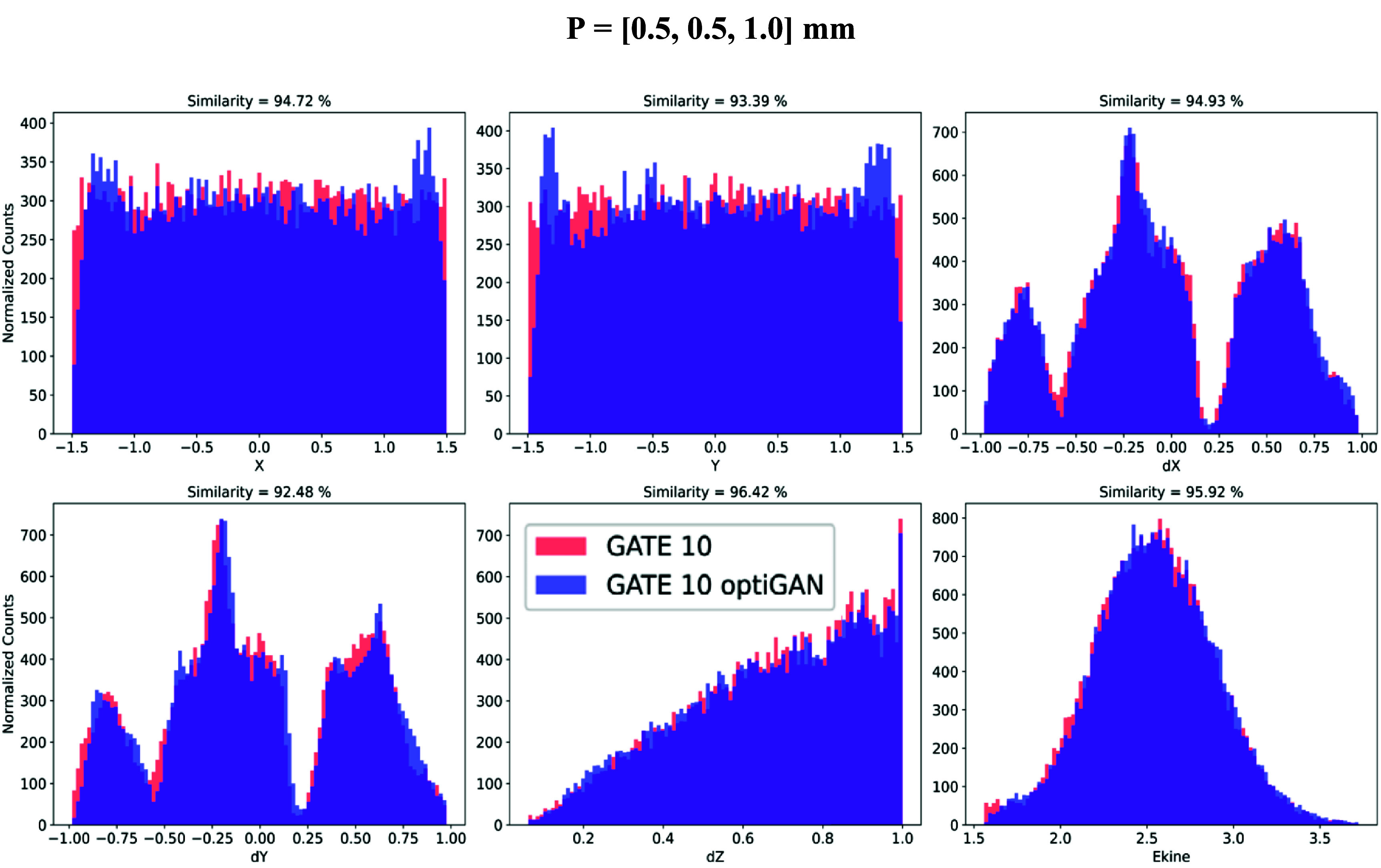
Comparison of GATE 10 and GATE 10–optiGAN simulations for optical photon transport. The plots show the normalized distributions of position (*X, Y*), direction (*dX, dY, dZ*) and kinetic energy for optical photons emitted at position *P* = [0.5, 0.5, 1.0]. GATE 10 results are shown in red and GATE 10–optiGAN in blue.

The high similarity between GATE 10 and the GATE 10–optiGAN simulations, with similarity exceeding ∼92% (figure 5), demonstrates that the GATE 10–optiGAN model successfully captures the complex dependencies between emission points and photon trajectories within the crystal, enabling the generation of synthetic optical photon transport data that closely reproduce conventional Monte Carlo simulation data. This validates the potential of optiGAN to be a computationally efficient alternative to traditional photon transport methods.

Notably, even for complex distributions such as the directional components that exhibit multiple peaks or highly skewed shapes (*dX, dY* and *dZ* in figure 5), GATE 10–optiGAN accurately replicated GATE 10 simulation photon distributions. This is particularly significant given that the distribution shapes can become highly intricate depending on the location of the emission point within the crystal (e.g. *dX* or *dY* distributions in figure 5).

**Figure 6.**
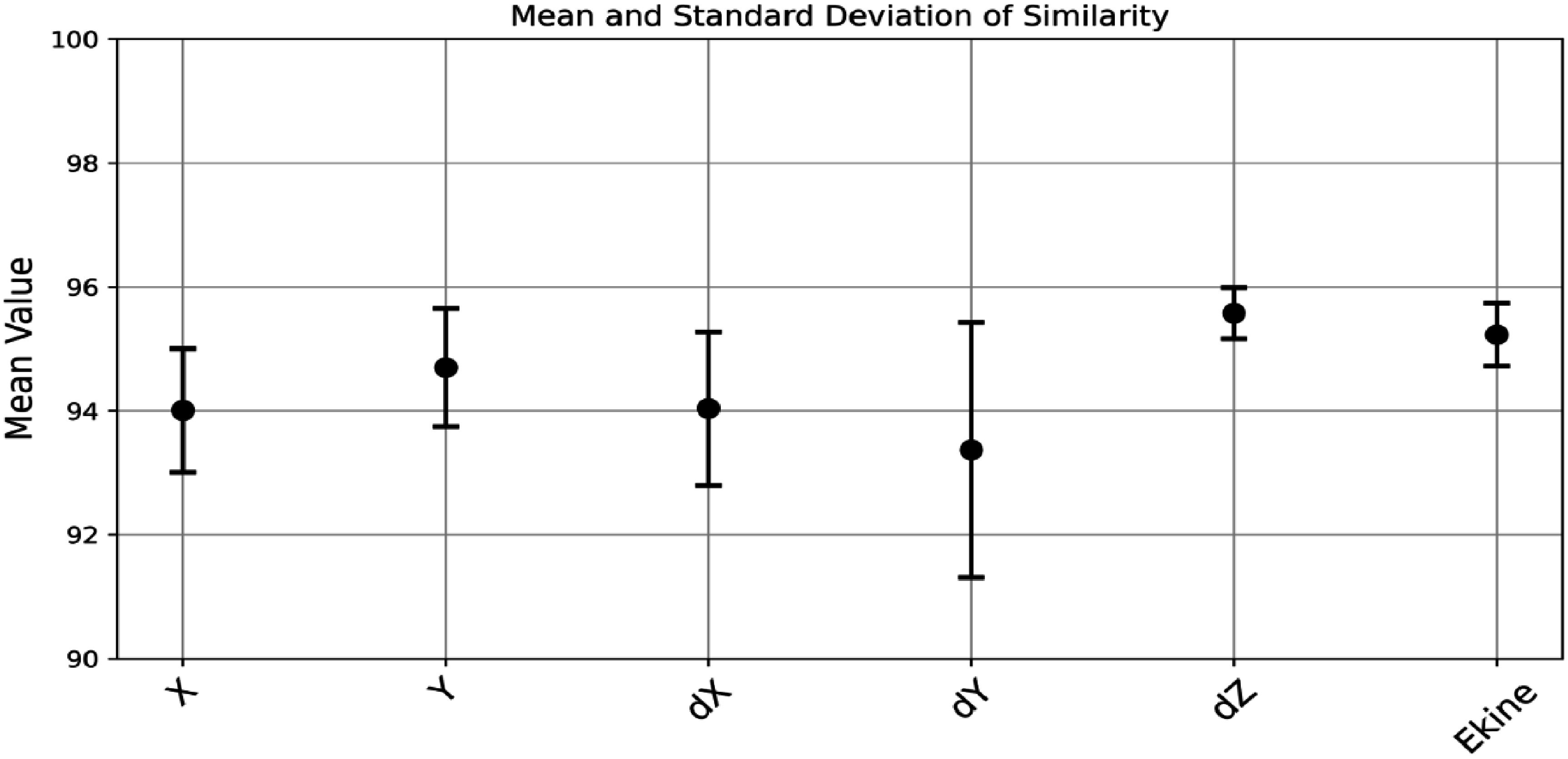
Mean and standard deviation of similarity between GATE 10 and GATE 10–optiGAN simulations for all emission coordinates within a 3 × 3 × 10 mm^3^ scintillation crystal.

Figure 6 presents the mean similarity scores along with their standard deviations for different optical photon transport parameters across all 30 emission coordinates. The results indicate consistently high similarity values, ranging between 92% and 97%. The observed variations in standard deviation for certain parameters (e.g. *X, Y, dX, dY* in figure 6) likely stem from the inherent randomness in GAN training, which may cause the model to fit certain emission coordinates more effectively than others. This does not indicate any issues with the integration process but rather suggests that the model could benefit from extended training or tuned hyperparameters to improve consistency.

### 3.3. Computational performance

**Figure 7.**
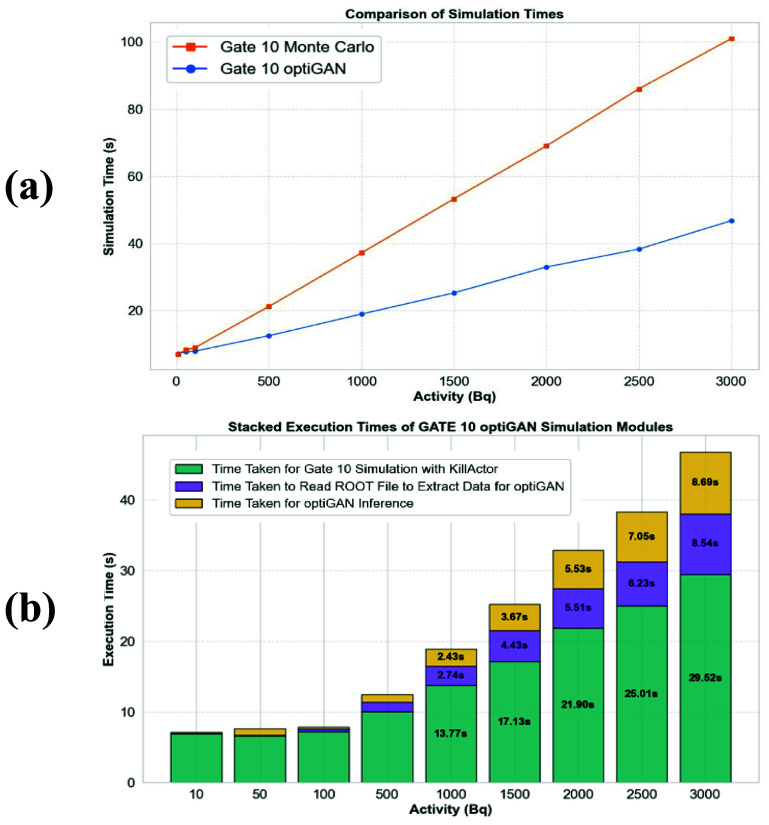
(a) Comparison of simulation times between GATE 10 (orange) and GATE 10–optiGAN simulations (blue). (b) Computational time analysis of the GATE 10-optiGAN pipeline using *py-spy*. This plot provides a detailed breakdown of the blue curve in (a), with different colored segments representing the execution time of various modules.

The comparison of simulation times between GATE 10–optiGAN and GATE 10 simulations, demonstrated an increasing computational cost gain with optiGAN with increasing activity (figure 7(a)). At lower activity levels (e.g. 10, 50, and 100 Bq), the difference in simulation times was relatively small, approximately 6%, while it reached 50% at 1000 Bq (simulation time of 18.9 s for GATE 10–optiGAN compared to 37.1 s with GATE 10). At the highest tested activity (3000 Bq), GATE 10–optiGAN achieved a nearly 60% reduction in simulation time (46.7 s with GATE 10–optiGAN compared to 1 min 41 s with GATE 10), highlighting its efficiency.

While the simulation time for both methods increased approximately linearly with activity, the rate of increase was lower for GATE 10–optiGAN, at 25% compared to 30% for GATE 10, indicating a better scaling of computational cost with higher activity levels.

A key observation from the analysis of GATE 10–optiGAN pipeline is that the major portion of the time was spent on the GATE 10 simulation with KillActor (section 2.3.2), which generated the ROOT file from which input for optiGAN is extracted. At higher activity levels (>1000 Bq), this process accounted for more than 60% of the total simulation time (teal color segment in figure 7(b)). Additionally, reading the generated ROOT file emerged as a major bottleneck, accounting for 15%–20% of the total execution time, with the percentage increasing as activity levels increase. (Purple color segment in figure 7(b)).

Since the ROOT file was necessary to extract the input data for optiGAN, its size grew with increasing activity levels, leading to longer readout times and impacting the overall execution time. For almost all activity levels, the time taken to read the ROOT file was comparable to the inference time required for optiGAN.

Regarding inference times (gold color in figure 7(b)), as activity levels increased, the number of optical photons also increased, resulting in a higher computational demand on the GPU for generating synthetic data. For example, at an activity level of 2000 Bq, approximately 4.8 million optical photons were generated, requiring 5.5 s for inference, whereas at 3000 Bq, this number increased to 7.2 million, taking 8.6 s.

As a result, the inference time scaled with the volume of generated output, yet it remained significantly lower than the Monte Carlo-based optical photon transport simulations.

It is also important to note that the inference stage in this context encompasses multiple steps beyond just optiGAN evaluation. It includes library loading, extraction of inputs from the ROOT file, generation of optical photon transport distributions using optiGAN, and saving the results as a ROOT file.

Library loading and input extraction from the ROOT file sometimes occurred in parallel or took less than 150 ms, while writing to the ROOT file happened in parallel during data generation.

## 4. Discussion and conclusion

In this work, the GAN based optical model, optiGAN, was successfully integrated into the new Python based version of GATE, GATE 10 and its results were validated against those obtained with GATE 9, which is widely used worldwide. The comparison confirmed both the accuracy and computational gain of the optiGAN integrated approach within the new GATE version. The work presented here also serves as a validation of optical simulations in GATE 10.

A key limitation identified in this study is the bottleneck associated with reading the ROOT file, as highlighted in figure 7(b). At this stage, ROOT files are essential for extracting useful information from GATE 10 and generating the input data for optiGAN. Since file reading and writing operations contributed significantly to the overall execution time, optimizing data handling techniques such as employing more efficient storage formats, parallelizing read/write, or using alternative methods for extracting the input data for optiGAN could further improve the efficiency of this approach. Addressing this limitation has the potential to ultimately make real-time or near-real-time optical photon transport simulations feasible.

Another important consideration is that simulation speed depends on the system’s hardware capabilities. A system equipped with a high-performance GPU will experience significantly faster optiGAN inference, further reducing overall simulation time. In general, any system with a GPU will achieve faster simulations using the GATE 10–optiGAN approach, as opposed to the Monte Carlo-based GATE 10 simulation, which relies on multi-threading on CPU but does not utilize the GPU for acceleration.

As discussed in section 2.3.2, the number of optical photons was determined using a *KillActor*, but this approach was not a definitive solution, as the goal was to eliminate the generation of optical photons. While *KillActor* helped reduce computational overhead, it remained a partial workaround rather than a definitive solution as it was required to count the number of emitted optical photons without modifying Geant4. Future research will aim at eliminating the need for both *KillActor* and optical photon generation entirely. One potential approach could involve incorporating a new feature in the GAN to estimate the number of detected optical photons per event position. Achieving this would lead to further improvements in computational efficiency, making GATE 10–optiGAN simulations even more scalable and time efficient.

Regarding the similarity metric, as discussed in section 2.4, JS distance was used as the evaluation methodology, applied through histogram-based estimation with 100 bins. Several tests revealed that the binning of the distributions affected the final similarity values, leading to differences of up to 2% (not shown).

The difference in results arise from inherent limitations of histograms, which rely on fixed binning and can introduce discretization artifacts, leading to a loss of fine-grained distributional details. The choice of bin width affected the results: too few bins led to an over-smoothed distribution, while too many bins led to overfitting noise rather than capturing the true data distribution. Additionally, fixed bin edges can arbitrarily split data points, distorting the actual shape of the underlying distribution.

Another contributing factor was the stochastic nature of the simulations. As shown in figure 3, even with an identical simulation setup, the results are not 100% identical due to this inherent variability. Ongoing tests aim to determine the extent to which these variations arise from by stochastic effects versus actual differences in the simulations.

Due to the continuous nature of GAN outputs, future work will also explore the use of kernel density estimation (KDE) and KDE-based JS distance to evaluate its robustness as a similarity metric for optiGAN generated distributions. Future work will continue to explore alternative methods for improving the comparison of distributions in optical photon transport simulations. Previous investigations into Wasserstein distance and Kolmogorov–Smirnov similarity revealed that these metrics often produced extremely high similarity scores, even in cases where the distributions visually did not align well. This discrepancy highlights the need for a more robust metric capable of accurately capturing both global and local differences in the data. Further investigations are ongoing to identify and develop more reliable evaluation methodologies.

One major limitation of optiGAN in its current form is its lack of generalizability. A dedicated training process is required for each specific simulation setup, including variations in crystal geometry, material composition, and photon energy. This implies that optiGAN models trained on one configuration (e.g. 3 × 3 × 10 mm^3^ BGO crystals) may not generalize well to others. Key factors affecting retraining include scintillator type, size, photodetector geometry, and emission spectrum. Consequently, the inference model is not yet portable across arbitrary detector configurations.

To address this and ensure broader adoption, future work will explore transfer learning techniques and model modularity to adapt trained networks to new setups with minimal retraining. Additionally, optimizing the training set by exploring strategies such as active sampling, data augmentation, or simulation-informed priors may reduce the amount of data required for new configurations. Understanding how these parameters impact convergence and accuracy will be essential to improving optiGAN’s applicability.

This study focused on a 3 × 3 × 10 mm^3^ BGO crystal; future work will extend the research to a broader range of scintillator geometries and compositions to assess optiGAN’s generalizability to different experimental configurations. Additionally, future research will explore transfer learning techniques to determine whether a model trained on one set of crystal configurations can be effectively adapted to others, potentially reducing the need for extensive retraining. These advancements will further validate and enhance the scalability of this approach. This is particularly significant for medical imaging applications, where large-scale Monte Carlo simulations are required for system modeling and optimization. One of the most prominent use cases is in PET scanner simulations, where accurate optical photon transport modeling plays a crucial role in detector response characterization, light collection efficiency estimation, and energy resolution analysis. By applying deep learning-based acceleration directly in GATE, researchers can conduct large-scale simulations that combine computational power with accurate physics modeling, facilitating faster experimental design, detector optimization, and real-time feasibility studies.

Moreover, this work highlights the potential of integrating deep learning techniques with Monte Carlo simulations to develop robust, physics-informed, and computationally efficient models for optical photon transport. As deep learning architectures continue to advance, exploring approaches such as physics-informed neural networks and adaptive generative modeling could provide promising avenues for further improvement.

This study serves as a foundation for future research aimed at bridging the gap between data-driven acceleration and physics-based simulations. By utilizing deep learning for efficient and scalable optical photon transport modeling, this work contributes to the development of computational methodologies that can significantly accelerate simulations across various scientific and industrial domains, including medical imaging, high-energy physics, and detector design.

The tools developed in this study are openly available on GitHub and integrated within GATE at: https://github.com/OpenGATE/opengate/tree/master/opengate/contrib/optical.

## Data Availability

The data that support the findings of this study are available upon reasonable request from the authors.

## References

[pmbade2b5bib1] Arjovsky M, Chintala S, Bottou L (2017). Wasserstein generative adversarial networks.

[pmbade2b5bib2] Berg E, Roncali E, Cherry S R (2015). Optimizing light transport in scintillation crystals for time-of-flight PET: an experimental and optical Monte Carlo simulation study. Biomed. Opt. Express.

[pmbade2b5bib3] Berg E, Roncali E, Kapusta M, Du J, Cherry S R (2016). A combined time-of-flight and depth-of-interaction detector for total-body positron emission tomography. Med. Phys..

[pmbade2b5bib4] Bert J (2012). Hybrid GATE: a GPU/CPU implementation for imaging and therapy applications.

[pmbade2b5bib5] Bert J, Perez-Ponce H, Bitar Z E, Jan S, Boursier Y, Vintache D, Bonissent A, Morel C, Brasse D, Visvikis D (2013). Geant4-based Monte Carlo simulations on GPU for medical applications. Phys. Med. Biol..

[pmbade2b5bib6] Buvat I, Lazaro D (2006). Monte Carlo simulations in emission tomography and GATE: an overview. Nucl. Inst. Methods Phys. Res. A.

[pmbade2b5bib7] de Oliveira L, Paganini M, Nachman B (2017). Learning particle physics by example: location-aware generative adversarial networks for physics synthesis. Comput. Softw. Big Sci..

[pmbade2b5bib8] Erdmann M, Glombitza J, Quast T (2019). Precise simulation of electromagnetic calorimeter showers using a Wasserstein generative adversarial network. Comput. Softw. Big Sci..

[pmbade2b5bib9] Fanelli C, Pomponi J (2020). DeepRICH: learning deeply Cherenkov detectors. Mach. Learn. Sci. Technol..

[pmbade2b5bib10] Geant4 Collaboration (2021). Physics reference manual. Version: geant4, 10.7. https://geant4-userdoc.web.cern.ch/UsersGuides/PhysicsListGuide/html/electromagnetic/emphyslist.html?highlight=g4emstandardphysics_option4.

[pmbade2b5bib11] Geant4 Physics List Group (n.d.). Physics List Guide. https://geant4-userdoc.web.cern.ch/UsersGuides/PhysicsListGuide/html/electromagnetic/emphyslist.html?highlight=g4emstandardphysics_option4.

[pmbade2b5bib12] Geant4 (n.d.). User Guide. https://geant4-userdoc.web.cern.ch/UsersGuides/PhysicsListGuide/html/electromagnetic/emphyslist.html?highlight=g4emstandardphysics_option4.

[pmbade2b5bib13] Goodfellow I J (2014). Generative adversarial nets.

[pmbade2b5bib14] Grevillot L (2020). GATE-RTion: a GATE/Geant4 release for clinical applications in scanned ion beam therapy. Med. Phys..

[pmbade2b5bib15] Gulrajani I, Ahmed F, Arjovsky M, Dumoulin V, Courville A (2017). Improved training of Wasserstein GANs.

[pmbade2b5bib16] Hashemi H, Hartmann N, Kuhr T, Ritter M (2023). PE-GAN: prior Embedding GAN for PXD images at Belle II. https://arxiv.org/abs/2303.00693.

[pmbade2b5bib17] Jan S (2004). GATE: a simulation toolkit for PET and SPECT. Phys. Med. Biol..

[pmbade2b5bib18] Jan S (2011). GATE V6: a major enhancement of the GATE simulation platform enabling modelling of CT and radiotherapy. Phys. Med. Biol..

[pmbade2b5bib19] Krah N (2024). GATE 10: a new versatile Python-driven Geant4 application for medical physics.

[pmbade2b5bib20] Mirza M (2014). Conditional generative adversarial nets. https://arxiv.org/abs/1411.1784.

[pmbade2b5bib21] openGATE (2024). openGATE collaboration. https://github.com/OpenGATE/opengate.

[pmbade2b5bib22] Paganini M, de Oliveira L, Nachman B (2018). CaloGAN: simulating 3D high energy particle showers in multilayer electromagnetic calorimeters with generative adversarial networks. Phys. Rev. D.

[pmbade2b5bib23] Roncali E, Cherry S R (2013). Simulation of light transport in scintillators based on 3D characterization of crystal surfaces. Phys. Med. Biol..

[pmbade2b5bib24] Roncali E, Mosleh-Shirazi M A, Badano A (2017a). Modelling the transport of optical photons in scintillation detectors for diagnostic and radiotherapy imaging. Phys. Med. Biol..

[pmbade2b5bib25] Roncali E, Stockhoff M, Cherry S R (2017b). An integrated model of scintillator-reflector properties for advanced simulations of optical transport. Phys. Med. Biol..

[pmbade2b5bib26] Santin G, Strul D, Lazaro D, Simon L, Krieguer M, Martins M V, Breton V, Morel C (2003). GATE: a Geant4-based simulation platform for PET and SPECT integrating movement and time management. IEEE Trans. Nucl. Sci..

[pmbade2b5bib27] Sarrut D (2014). A review of the use and potential of the GATE Monte Carlo simulation code for radiation therapy and dosimetry applications. Med. Phys..

[pmbade2b5bib28] Sarrut D (2021a). Advanced Monte Carlo simulations of emission tomography imaging systems with GATE. Phys. Med. Biol..

[pmbade2b5bib29] Sarrut D, Etxebeste A, Krah N, Létang J M (2021b). Modeling complex particles phase space with GAN for Monte Carlo SPECT simulations: a proof of concept. Phys. Med. Biol..

[pmbade2b5bib30] Sarrut D, Krah N, Létang J M (2019). Generative adversarial networks (GAN) for compact beam source modelling in Monte Carlo simulations. Phys. Med. Biol..

[pmbade2b5bib31] Srikanth A, Trigila C, Roncali E (2024). GPU optimization techniques to accelerate optiGAN—a particle simulation GAN. Mach. Learn. Sci. Technol..

[pmbade2b5bib32] Strulab D, Santin G, Lazaro D, Breton V, Morel C (2003). GATE (Geant4 application for tomographic emission): a PET/SPECT general-purpose simulation platform. Nucl. Phys. B.

[pmbade2b5bib33] Trigila C, Mehadji B, Roncali E (2024). Inter-Crystal optical crosstalk in a crystal array.

[pmbade2b5bib34] Trigila C, Moghe E, Roncali E (2021). Standalone application to generate custom reflectance Look‐Up Table for advanced optical Monte Carlo simulation in GATE/Geant4. Med. Phys..

[pmbade2b5bib35] Trigila C, Srikanth A, Roncali E (2023). A generative adversarial network to speed up optical Monte Carlo simulations. Mach. Learn. Sci. Technol..

